# Psychological distress in South Africa: Analysis of 2017 national household survey data

**DOI:** 10.4102/hsag.v30i0.2920

**Published:** 2025-09-23

**Authors:** Shandir Ramlagan, Inbarani Naidoo, Alicia North, Sean Jooste

**Affiliations:** 1 Faculty of Public Health, Societies and Belongings, Human Sciences Research Council, Pretoria, South Africa; 2 Faculty of Public Health, Societies and Belongings, Human Sciences Research Council, Durban, South Africa; 3 Research Centre for Palliative Care, Death and Dying, Flinders University, Adelaide, Australia; 4 Faculty of Public Health, Societies and Belongings, Human Sciences Research Council, Cape Town, South Africa

**Keywords:** psychological distress, South Africa, K-10, HIV status, women

## Abstract

**Background:**

The prevalence of psychological distress in South Africa requires updated estimates.

**Aim:**

This article aims to determine the prevalence of and factors associated with non-specific psychological distress at a national level in South Africa in 2017.

**Setting:**

The study utilised data from a 2017 nationally representative, cross-sectional, population-based household survey.

**Methods:**

Interviews were conducted with 36 609 individuals, aged ≥15 years. Prevalence of psychological distress, using the Kessler-10 scale, was varied by demographics, health status, substance use and human immunodeficiency virus (HIV). Chi-square tests, univariate analyses and a multivariate logistic model were constructed. Collinearity between independent variables was assessed.

**Results:**

Almost half (47%) of the respondents could be classified as psychologically distressed. Higher levels of psychological distress were observed for women (52.6%), among those aged 25 years – 49 years (53.8%), black Africans (84.2%), those with secondary school level of education (64.1%), unmarried people (73.7%) and those residing in urban areas (63.5%). The multivariate logistic regression model found significant differences for sex, self-reported health status, alcohol use, employment status and locality. Human immunodeficiency virus serostatus did not play a major role in psychological distress.

**Conclusion:**

Special attention should be paid to women in the age group of 25 years – 49 years, the unemployed, those with poor general health and people living in urban areas to address the high prevalence of psychological distress in South Africa.

**Contribution:**

This study adds to the literature on the psychological distress amongst those living in South Africa.

## Introduction

There is a growing global shift for the need to address mental health issues, with an increased focus on prevention as well as improving treatment for more common mental disorders such as psychological distress (South African Health Review [SAHR] 2015; Wainberg et al. 2017). Psychological distress, which includes anxiety and depression, is a state of emotional suffering that presents with various characteristics such as fluctuating moods, loss of interest, feeling of helplessness and dysfunction, feeling anxious and loss of hope (Mathew et al. 2016).

There are a range of factors associated with psychological distress, including: adverse socio-economic conditions; stressful social and family environment; life events; academic stress; working conditions; socio-cultural factors such as gender; lower educational attainment; a lack of social support; and stressful life events (Mthembu et al. 2017; Ramlagan et al. 2024; Talala et al. 2011). Factors that have contributed to the neglect of mental health issues in the past, such as stigma against those living with mental illness, myths around mental issues and ignorance as to the extent of mental health problems, may continue to hinder current efforts to mitigate mental health issues (Lund 2017). It is important to notice that distress does not equate with psychiatric disorder, although it may contribute to it. Psychological distress is a common occurrence globally where an estimated 542 million people are living with depression or anxiety symptoms, representing an increase of more than 18% from 2005 to 2015 (Islam 2019). Furthermore, data from a meta-analysis of 174 surveys conducted across 63 countries between the years 1980 and 2013 reported a 17.6% prevalence of common mental disorders (Islam 2019; Steel et al. 2014). In addition, research studies that have been conducted in African countries have shown that individual, socio-cultural and socio-demographic factors are strong influences in the response to mental disorders and illness (Mthembu et al. 2017; Ramlagan et al. 2024).

Several studies conducted across Africa have found varying prevalence of psychological distress. A study conducted in Ghana found nearly 20% of men and women reporting cases of moderate or severe psychological distress (Gust et al. 2017). A household survey conducted in the Nyanza province of Kenya reported a 10.8% prevalence of common mental disorders such as anxiety and/or depression, panic disorder and generalised anxiety disorder (Gust et al. 2017). The prevalence of psychological distress and mental disorders continues to escalate, and South Africa faces a growing burden of mental and neurological disorders, which often co-occurs with human immunodeficiency virus (HIV) and other chronic diseases (Kaminer, Owen & Schwartz 2018). To date, the South African Stress and Health (SASH) study conducted between 2002 and 2004 was the only national representative study that has provided data on the prevalence of common mental disorders (Herman et al. 2009). Lifetime prevalence of common mental disorders was 30.3% (Herman et al. 2009).

Later studies showed a similar trend to the findings of SASH. In 2012, a national study on the health and nutrition of South African adults found that the 28.4% of participants reported psychological distress, varying from 10.3% reporting moderate levels of distress, 4.2% reporting high levels of distress and 2.2% reporting very high levels of distress (Mthembu et al. 2017; Shisana et al. 2013). In the same year that the health and nutrition survey was conducted, a national household survey on the prevalence and incidence of HIV was conducted (Shisana et al. 2013) and from that data, 23.9% of the participants reported psychological distress (Mthembu et al. 2017). Females reported significantly higher levels of psychological distress than their male counterparts (Mthembu et al. 2017; Shisana et al. 2014; Simbayi et al. 2019; Ramlagan et al. 2024).

Mental illness is a major risk factor for HIV infection, as it not only impairs an individual’s judgement of a situation but also increases high-risk-taking behaviour (Fang, Chuang & Al-Raes 2019). For individuals that have been diagnosed with HIV, mental health presentations frequently include co-occurring conditions such as depression, anxiety, alcohol abuse and post-traumatic stress disorder (PTSD) (Kagee et al. 2017). Adherence to medication, more specifically antiretroviral treatment, is often poor because of individuals suffering from mental disorders (HIV Clinicians Society 2013). According to Mthembu et al. (2017), stressful life events have been identified as a significant risk factor for psychological distress, and the interplay between socio-demographic characteristics and stress-related factors may further heighten vulnerability to such distress. Additional factors include HIV-related risks such as sexual behaviours and alcohol use, multiple sexual partners, transactional sex and inconsistent condom use (Mthembu et al. 2017; Shisana et al. 2014; Simbayi et al. 2019).

Psychological distress has not been well studied and very few research studies have been conducted at a national level in South Africa. Psychological distress needs to be investigated at regular intervals, especially in developing countries in order to gain a better understanding of the prevalence as well as to inform interventions that need to be implemented. The aim of this article is to add to the literature on factors associated with non-specific psychological distress at a national level in South Africa, and the objectives are to determine: (1) the prevalence of non-specific psychological distress, which was measured by the Kessler (K-10) psychological distress scale and (2) factors associated with non-specific psychological distress at a national level.

## Research methods and design

### Study design

This study uses data gathered during a cross-sectional, national, population-based household survey, which was conducted between December 2016 and February 2018. The national study used a multi-stage stratified random cluster sampling approach that is explained elsewhere (Simbayi et al. 2019). Briefly, 1000 small area layers (SAL) were selected from the 2015 national population sampling frame through stratified, disproportionate sampling. In each SAL, 15 households were randomly selected and in each household, all household members of all ages, who resided in that household the previous night, were eligible to participate. The survey encompassed household members living in hostels and private residences throughout South Africa, but excluded individuals residing in educational institutions, retirement homes, hospitals and uniformed-service barracks.

Once the correct sampled household was identified on the ground, a fieldwork supervisor accompanied by a fieldworker entered the household and introduced themselves and the study to the delegated authority figures in the household. Permission was sought from the delegated authority to conduct the survey in the selected household. The questionnaire was interviewer-administered and electronically captured by fieldworkers using a Mercer A105 tablet enabled with free CSPro software (Simbayi et al. 2019). Once the questionnaire was completed, and if consent was given, a blood sample was collected from the respondent. The blood sample and questionnaire were barcoded to allow linkage for data analysis.

A total of 9656 households agreed to participate, 36 609 individuals agreed to be interviewed and 23 910 provided a blood sample for HIV testing thereby giving a household, individual and blood sample response rate of 82.2%, 93.6% and 61.1%, respectively (Simbayi et al. 2019).

### Measures

The main dependent variable of this study, non-specific psychological distress, was derived from the 10 item Kessler psychological distress scale (K10) (Kessler et al. 2002). Respondents were asked about their feelings over the last 30 days. Questions in the scale included but were not limited to: ‘During the last 30 days, about how often did you feel tired out for no good reason?’; ‘During the last 30 days, about how often did you feel hopeless?’; and ‘During the last 30 days, about how often did you feel depressed?’ Responses were recorded on a 5 point Likert scale in which none of the time = 1, a little of the time = 2, some of the time = 3, most of the time = 4, and all the time = 5. The scores for all questions are summed where higher scores reflect higher level of psychological distress (Kessler et al. 2002). Similar to a previous study among the general population in South Africa (Mthembu et al. 2017), the scale was dichotomised into two categories, where a total score of 20 or below indicated no psychological distress (coded as 0), while a score above 20 indicated mild to severe psychological distress (coded as 1) (Andrews & Slade 2001).

### Socio-demographic characteristics

Socio-demographic variables included age (15 years – 24 years, 25 years – 49 years and ≥ 50 years), race (black African people and ‘others’ including white people, mixed race and Indian people or Asians), sex (male, female), living arrangements (living with husband or wife, not living with husband or wife, living with boyfriend or girlfriend or civil union partner, in a steady relationship but not living together, single or not in a steady relationship), education (none, primary, secondary, tertiary), employment (unemployed, employed), province of residence and geotype (urban, rural informal, rural). It is important to note that the race classifications mentioned above are part of South Africa’s apartheid history and remain relevant today. In this race classification, ‘mixed race’ refers to a distinct race group of mixed European (‘white’) and black, Indian or Asian ancestry. Although the authors do not agree with such race classifications, it is included in the analysis in order to ensure the injustices of the past apartheid government are corrected. ‘Rural informal’ is defined as including tribal authority areas as designated in South Africa, while ‘rural’ only includes farming areas.

### Health-related characteristics

Respondents were asked about their health status and reported whether they currently believed their health to be poor, fair, good or excellent. Respondents were also asked if they had any medical aid or a medical benefit scheme (yes, no). For substance use, this study utilised the Alcohol Use Disorder Identification Test (AUDIT) (National Institute of Alcohol Abuse and Alcoholism [NIAAA] 2015) and followed the methodology of Peltzer, Davids and Njuho (2011), who also utilised it in a nationally representative sample in South Africa. The alcohol AUDIT score was derived from 10 questions aimed to assess the frequency and severity of alcohol consumption with abstainers and low risk drinkers scoring from 1 to 7, high risk or harmful drinkers scoring from 8 to 19 and hazardous drinkers scoring 20+.

In terms of sexual health, respondents were asked for the number of sexual partners in the last 12 months (dichotomised as one partner, two or more partners), and if their current partner’s age was within 5 years of their own age, younger by 5 years or older by 5 years. Condom use was assessed as being used during the last sex (yes, no) and consistency of condom use (yes, used a condom every time or no). For HIV measures, respondents were asked if they ever had an HIV test (yes, no), and if they received the results of the HIV test (yes, no). Human immunodeficiency virus status was measured using dry blood spots (DBS) taken from respondents who consented. The DBS samples were obtained *via* finger-prick and tested for HIV-antibodies using an algorithm involving three distinct enzyme immunoassays (Simbayi et al. 2019). Results were interpreted as sero-positive or sero-negative.

### Data analysis

The outcome variable was the presence and/or absence of psychological distress. All variables were coded as described above and weighted data were analysed in Stata Version 15.1. Chi-square tests were used to assess the burden of psychological distress stratified by each socio-demographic and health variable reporting column counts and proportions with 95% confidence intervals and significance at *p* = 0.05. Weighted percentages are presented in Table 1 and Table 2. Thereafter, a multivariate logistic model was constructed using variables found to be significant in the univariate analyses and collinearity between independent variables was assessed.

**TABLE 1:** Reported psychological distress by socio-demographic characteristics among those aged 15 years and older, South Africa, 2017.

Variables	*n*	%†	95% CI	*p*-value
**Sex**				
Male	8011	47.4	46.4–48.4	< 0.001
Female	9579	52.6	51.6–53.6	
**Age (years)**				
15–24	1397	21.4	20.0–22.9	< 0.001
25–49	2821	53.8	51.9–55.7	
50+	1895	24.8	23.1–26.5	
**Race**				
Black African	12 622	84.2	82.2–86.0	< 0.001
Other	4968	15.8	14.0–17.8	
**Marital status**				
Married	1429	26.7	24.7–28.8	0.010
Unmarried	3447	73.3	71.2–75.3	
**Current living arrangement**				
Living with husband or wife	1288	23.9	22.0–25.9	0.060
Living on own or other arrangement but not living with husband	675	15.6	13.6–17.9	
Living together with boyfriend or girlfriend or civil union partner	328	7.6	6.3–9.0	
Single or divorced or widowed or in a steady relationship but not living together	865	18.5	16.5–20.7	
Single; not in a steady relationship	1687	34.5	32.2–36.8	
**Education**				
No education	28	0.8	0.4–1.5	< 0.001
Primary	1106	23.4	21.4–25.6	
Secondary	2291	64.1	61.9–66.3	
Tertiary	400	11.7	10.1–13.5	
**Employment**				
Unemployed	3383	69.4	67.1–71.6	< 0.001
Employed	1409	30.6	28.4–32.9	
**Province**				
Western Cape	1765	9.7	8.1–11.4	< 0.001
Eastern Cape	1981	12.2	10.4–14.2	
Northern Cape	1612	2.4	1.8–3.2	
Free State	1331	5.2	4.1–6.5	
KwaZulu-Natal	3808	20.0	16.9–23.6	
North West	1684	7.8	6.5–9.5	
Gauteng	2202	23.9	20.5–27.7	
Mpumalanga	1879	8.6	7.0–10.4	
Limpopo	1328	10.3	8.6–12.2	
**Geotype**				
Urban	10 773	63.5	59.1–67.6	< 0.001
Rural informal (tribal areas)	5026	32.3	28.2–36.7	
Rural (farms)	1791	4.2	3.0–5.7	

† , Weighted percentages.

### Ethical considerations

Ethical approval for the survey was granted by the Human Sciences Research Council (HSRC) Research Ethics Committee (REC) with protocol approval number REC: 4/18/11/15. Approval was also granted by the Associate Director for Science, Center for Global Health (CGH), Centers for Disease Control and Prevention (CDC). Once overall permission was granted for an interview to occur, eligible household members completed a written informed consent form individually and in private with the fieldworker before going on to complete a corresponding, age appropriate questionnaire.

## Results

In Table 1, the authors report on psychological distress (Cronbach’s alpha reliability, α = 0.93) in South Africa by socio-demographic characteristics among those aged 15 years and older for 2017. Overall, just under half (46.7%) of the respondents (*n* = 36 470) could be classified as psychologically distressed. Findings of the univariate analyses show significant differences (*p* < 0.001) in psychological distress among sex, age, race, marital status, education levels and employment status. There were also significant differences in psychological distress based on geographic location by province and geotype. Significantly higher levels of psychological distress (*p* < 0.001) were observed for women (52.6%), among those aged 25 years – 49 years (53.8%), black Africans (84.2%), those with secondary school level of education (64.1%) and unmarried people (73.7%) (Table 1). In terms of provincial stratification, people living in Gauteng (23.9%) and KwaZulu-Natal (20.0%) provinces made up just under half of all those with psychological distress. Furthermore, significantly more psychologically distressed people lived in urban areas (63.5%) compared to rural areas.

Analysis of health-related factors associated with psychological distress showed significant differences for self-reported health status, medical aid membership, sexual age mixing, alcohol AUDIT score and HIV testing and status (see Table 2). About half (49.6%) of all respondents indicated that they were in good health, while the majority (88.3%) indicated they did not belong to a medical benefits insurance scheme that would allow them utilise private health care and have some or all of that cost absorbed by the insurance. A total of 25.5% of people whose sexual partners were aged 5 years more and older and 54.8% whose sexual partners were aged within 5 years of the individual or younger indicated they were distressed. A comparison of mental health among alcohol drinkers showed significant differences between the abstainers and drinkers, with a significant majority of abstainers (78.8%) indicating they were distressed.

**TABLE 2:** Reported psychological distress by health characteristics among those aged 15 years and older, South Africa, 2017.

Variables	*n*	%†	95% CI	*p*-value
**Self-reported health status**				
Excellent	5930	36.6	34.9–38.4	< 0.001
Good	7636	49.6	47.8–51.3	
Fair	1611	10.8	10.0–11.7	
Poor	443	3.0	2.6–3.4	
**Have medical aid or medical benefit scheme**				
Yes	576	11.7	9.9–13.7	< 0.001
No	4155	88.3	86.3–90.1	
One partner	1934	88.7	86.2–90.7	0.493
Two or more partners	212	11.3	9.3–13.8	
**Age mixing**				
5+ years older	570	25.5	23.3–27.8	< 0.001
5+ years younger	379	19.8	17.4–22.4	
Within 5 years older or younger	1176	54.8	51.7–57.8	
**AUDIT score** ‡				
Abstainers	5156	78.8	76.7–80.7	< 0.001
Low risk drinkers (1–7)	685	11.8	10.5–13.3	
High risk drinkers (8–19)	387	7.6	6.5–8.8	
Hazardous drinkers (20+)	103	1.8	1.4–2.4	
**Condom use at last sex**				
No	1371	61.1	57.9–64.1	0.980
Yes	772	38.9	35.9–42.1	
**Consistency of condom use**				
No	2109	98.7	97.6–99.3	0.728
Yes	16	1.3	0.7–2.4	
**Ever had an HIV test**				
Yes	6924	48.2	46.4–50.0	< 0.001
No	8215	51.8	50.0–53.6	
**Received HIV test results**				
Yes	3622	95.5	94.5–96.3	0.014
No	195	4.5	3.7–5.5	
**HIV serostatus**				
Positive	1055	11.2	10.4–12.2	< 0.001
Negative	10 177	88.8	87.8–89.6	

HIV, human immunodeficiency virus; AUDIT, alcohol use disorder identification test.

† , Weighted percentages.

‡ , Cronbach’s alpha reliability for AUDIT, α = 0.83.

Among psychologically distressed people, a slight majority (51.8%) indicated they had never tested for HIV. However, among distressed people, a significant majority self-reported they were aware of their HIV status (95.5%, *p* < 0.001). Furthermore, a significant majority were found to be HIV negative (88.8%, *p* < 0.001). Being both distressed and HIV positive was indicated by 11.2% of the respondents.

Results of the multivariate logistic regression model found significant differences in psychological distress for sex, self-reported health status and alcohol use based on the AUDIT score, employment status, locality and province (Table 3). A test for collinearity between independent variables used in the model found no correlation with a mean variance inflation factor (VIF) = 1.19 (range: 1–1.44). Our results suggest that psychological distress is gendered, in that women were twice as likely to be psychologically distressed than men (adjusted odds ratio [AOR] 2.0, 95% CI 1.51–2.54; *p* < 0.001). There was strong evidence to suggest that self-rated excellent health status also played a significant role in determining distress. Those with good, fair or poor health were more likely to suffer distress compared to those with excellent health (*p* < 0.001). Furthermore, those with poor health were nearly six times more likely to be distressed compared to those with excellent health (AOR 5.6, 95% CI 3.22–9.89; *p* < 0.001).

**TABLE 3:** Multivariable model for psychological distress showing significant variables, South Africa, 2017.

Variables	AOR	95% CI	*p*-value
**Sex**			
Male	Ref	-	-
Female	1.96	1.51–2.54	< 0.001
**Age (years)**			
15–24	1.11	0.74–1.67	0.618
25–49	1.10	0.81–1.49	0.537
50+	Ref	-	-
**Race**			
Black African people	1.08	0.75–1.55	0.686
Other	Ref	-	-
**Marital status**			
Unmarried	1.09	0.84–1.40	0.524
Married	Ref	-	-
**Education**			
No education	4.84	0.65–36.36	0.125
Primary	1.15	0.80–1.65	0.465
Secondary	0.89	0.65–1.24	0.498
Tertiary	Ref	-	-
**Employment status**			
Unemployed	1.39	1.14–1.71	0.001
Employed	Ref	-	-
**Province**			
Western Cape	1.01	0.64–1.60	0.962
Eastern Cape	1.29	0.84–2.00	0.249
Northern Cape	1.65	1.02–2.65	0.040
Free State	1.29	0.85–1.97	0.228
North West	2.17	1.47–3.22	< 0.001
Gauteng	1.63	1.10–2.41	0.015
Mpumalanga	2.97	1.95–4.54	< 0.001
Limpopo	1.09	0.68–1.76	0.717
KwaZulu-Natal	Ref	-	-
**Geotype**			
Urban	1.37	1.05–1.80	0.023
Rural (farms)	0.73	0.44–1.21	0.219
Rural informal (tribal areas)	Ref	-	-
**Self-reported health status**			
Excellent	Ref	-	-
Good	1.56	1.22–1.99	< 0.001
Fair	2.89	2.11–3.96	< 0.001
Poor	5.64	3.22–9.89	< 0.001
**Have medical aid or medical benefit scheme**			
Yes	1.01	0.64–1.58	0.971
No	Ref	-	-
**Age mixing**			
5+ years older	0.96	0.77–1.20	0.729
5+ years younger	1.14	0.86–1.51	0.356
Within 5 years older or younger	Ref	-	-
**AUDIT score**			
Abstainers	Ref	-	-
Low risk drinkers	0.88	0.66–1.18	0.397
High risk drinkers	1.36	0.96–1.94	0.086
Hazardous drinkers	2.88	1.39–5.98	0.005
**Tested for HIV and received results**			
Yes	Ref	-	-
No	0.99	0.60–1.66	0.984
**HIV serostatus**			
Negative	Ref	-	-
Positive	1.14	0.93–1.41	0.210

HIV, human immunodeficiency virus; AUDIT, alcohol use disorder identification test.

Results show that hazardous drinking is a significant risk factor for being distressed, with nearly three times as many hazardous drinkers (AOR 2.9, 95% CI 1.4–6.0, *p* = 0.005) being distressed compared to abstainers. Unemployed respondents were more likely to be psychologically distressed than their employed counterparts (AOR 1.4, 95% CI 1.14–1.71; *p* = 0.001). Those living in urban areas were more likely to be distressed compared to their rural counterparts (AOR 1.4, 95% CI 1.04–1.80; *p* = 0.023). The authors noticed provincial differences as well; people living in the Northern Cape, North West, Gauteng and Mpumalanga provinces were more likely to be distressed than those living in KwaZulu-Natal (Table 3). The results also suggest that HIV serostatus did not play a major role in psychological distress.

## Discussion

The study sought to update the estimates of non-specific psychological distress among those 15 years and older in South Africa and determine the factors associated with non-specific psychological distress at a national level. Using a nationally representative sample and a 10-item psychological distress (K10) scale (Kessler et al. 2002), this study found that in 2017, almost half of the respondents could be classified as psychologically distressed. This is exceptionally high given that in 2012, two separate South African national studies, utilising the K10 scale, found that prevalence of psychological distress was 24% (Mthembu et al. 2017) and 28% (Shisana et al. 2013). This drastic increase over the course of 5 years is of great concern and needs to be alleviated immediately.

The study also found significantly higher levels of psychological distress among the black African segment of the population as well as among women. This finding is similar to that of the 2012 nationally representative survey (Mthembu et al. 2017) and shows that over the last 5 years, progress has not been made to mitigate psychological distress among these groups. It is important to observe that the 2012 survey utilised similar variables and the same psychological distress cut-off scores as this 2017 study (Mthembu et al. 2017). This distress would be a remnant of South Africa’s apartheid past where the disenfranchisement of non-whites led to racial disparities in mental health (Jackson et al. 2010). The multivariable logistic regression results show that psychological distress is gendered, where women in South Africa were twice as likely to be psychologically distressed as men. These results are consistent with literature that informs that women in general report more psychological distress than men (Mthembu et al. 2017; Shisana et al. 2014; Smith et al. 2018; Ramlagan et al. 2024). The increased distress exhibited by women could also be explained by patriarchal ideology, lower education, less access to income generation, abuse and poorer health (Islam 2019; Mthembu et al. 2017), all of which are beyond the scope of this article and will require further study.

Psychological distress also increased in 2017 among those aged 25 years – 49 years when compared to the 2012 survey that reported higher levels of psychological distress among those 50 years and older (Mthembu et al. 2017). This is of concern as those in the 25–49-year-old age group make up the majority of the employed sector in South Africa. This study also found that unemployed respondents were more likely to be psychologically distressed than their employed counterparts, which could explain why psychological distress has increased in this age group.

Provincially, psychological distress was found to be more prevalent in North West, Gauteng, Mpumalanga and Northern Cape. In a briefing on the state of mental health services in the Northern Cape, challenges such as the need for a district mental health specialist team, mental health capacity and the shortage of psychiatrists were emphasised (Northern Cape Department of Health 2017). The Northern Cape briefing was further stressed in findings from a national survey, which showed that the lack of mental health human resources, infrastructure and medication supply could be reasons for the constrained mental health services across all provinces in South Africa (Docrat et al. 2017).

The finding that those with poor health were more likely to be psychologically distressed compared to those with excellent health and that those with psychological distress were more likely to report poor health was expected (St-Pierre et al. 2019). Poor overall health and poor psychological distress are interrelated (Islam 2019; Vancampfort et al. 2017), as well as the finding that hazardous drinking was a significant risk factor for being psychologically distressed, which could be related to self-medication with alcohol by persons with some mental health disorders (Bolton, Robinson & Sareen 2009; Leeies et al. 2010). Those living in urban areas were more likely to be psychologically distressed when compared to their rural counterparts. This finding is similar to that of Islam (2019) who had similar results in Bangladesh, but contradictory to findings in the United States of America (Basta, Shacham & Reece 2009; Hoyt et al. 1997). These contradictions need to be better understood and thus require further investigation.

### Study limitation

The study relied on self-reported psychological distress and may thus be subject to bias.

## Conclusion

The prevalence of psychological distress in South Africa has increased over the past 5 years. Strategies need to be developed that mitigate psychological distress in the general population, with special attention being paid to women, those between the age group of 25 years – 49 years, those unemployed, those with poor general health and those living in urban areas. In order to develop these strategies, further investigation is required at a national level on mental health in South Africa, as well as to identify the distress for different people.
